# A qualitative study on the stigma experienced by people with mental health problems and epilepsy in the Philippines

**DOI:** 10.1186/s12888-018-1902-9

**Published:** 2018-10-05

**Authors:** Chika Tanaka, Maria Teresa Reyes Tuliao, Eizaburo Tanaka, Tadashi Yamashita, Hiroya Matsuo

**Affiliations:** 1 Graduate School of Health Sciences, Kobe University, 701, 2-6-2, Yamamoto-dori, Chuo-ku, Kobe, Hyogo 650-0003 Japan; 2 City Health Office, City Government of Muntinlupa, Muntinlupa, Philippines; 3 Hyogo Institute for Traumatic Stress, Kobe, Japan; 4 Kobe City College of Nursing, Kobe, Japan

**Keywords:** Stigma, Discrimination, Mental illness, The Philippines, Qualitative

## Abstract

**Background:**

Stigma towards people with mental health problems (PMHP) is known to have substantial negative impacts on their lives. More in-depth exploration of the stigma and discrimination experienced by PMHP in low- and middle-income countries is needed. Previous research suggests that negative attitudes towards PMHP are widespread among the Filipino general public. However, no study has investigated PMHP’s own experiences of being stigmatised in the Philippines.

**Methods:**

A qualitative study was conducted on the stigma experienced by PMHP (including people with epilepsy) and its related factors in the Philippines, employing the constructivist grounded theory approach. We analysed data on 39 PMHP collected through interviews with PMHP, their carers, and community health volunteers who know them well.

**Results:**

The findings highlight the culturally and socio-economically specific contexts, consequences, and impact modifiers of experiences of stigma. Participants emphasised that PMHP face stigma because of the cultural traits such as the perception of mental health problem as a disease of the family and the tendency to be overly optimistic about the severity of the mental health problem and its impact on their life. Further, stigma was experienced under conditions where mental health care was not readily available and people in the local community could not resolve the PMHP’s mental health crisis. Stigma experiences reduced social networks and opportunities for PMHP, threatened the economic survival of their entire family, and exacerbated their mental health problems. An individual’s reaction to negative experiences can be fatalistic in nature (e.g. believing in it is God’s will). This fatalism can help PMHP to remain hopeful. In addition, traditional communal unity alleviated some of the social exclusion associated with stigma.

**Conclusions:**

The study indicates that existing stigma-reduction strategies might have limitations in their effectiveness across cultural settings. Therefore, we propose context-specific practical implications (e.g. emphasis on environmental factors as a cause of mental health problems, messages to increase understanding not only of the possibility of recovery but also of challenges PMHP face) for the Philippines.

**Electronic supplementary material:**

The online version of this article (10.1186/s12888-018-1902-9) contains supplementary material, which is available to authorized users.

## Background

Stigma and discrimination against people with mental health problems (PMHP) are a global public health issue [1–3] and can have substantial negative impacts on all aspects of a person’s life, from employment and housing to social and family life [4–7]. Public stigma, the general public’s reaction towards a stigmatised group, can be conceptualised as having three distinct elements [8]. First, a negative belief about a stigmatised group is seen as stereotype. Second, an emotional reaction to the stereotype is seen as prejudice. Third, a behavioural manifestation of the prejudice is discrimination. Historically, research on stigma related to mental health has been conducted mainly on stereotypes, prejudices, and intentions to discriminate that are held by the general public with regard to PMHP. Such research revealed that the general public frequently label PMHP as dangerous, blameworthy, incompetent and weak, which is often accompanied with emotions of fear and anger and can lead to behavioural intention of avoidance, punishment, and coercion [9–12]. Further, the literature shows that internalisation of public stigma or self-stigma is also frequent among PMHP, which reduces self-esteem, causes social isolation, and inhibits help-seeking behaviour [6, 13–15].

Recent research has more often investigated levels of discrimination using direct reports from PMHP. The results of such research suggest that discrimination against PMHP is a universal phenomenon around the world [2, 3, 16]; however, PMHP’s experiences of discrimination and its related factors might differ in high-income countries (HICs) versus low- and middle- income countries (LMICs). Some studies suggest that PMHP experience a lower level of stigma in LMICs [17], such as India [18], China [19], and Nigeria [20], compared with HICs. The reasons for the more positive acceptance of PMHP in those settings have been considered to be a more supportive environment with social cohesion as well as more social role options that PMHP are able to fulfil [21, 22]. At the same time, there is also accumulating evidence revealing that in LMICs, experiences of stigma, discrimination and human rights abuses related to mental health problems are common and severe [23–27]. The stigmatisation in LMICs has been attributed to the combined effects of socioeconomic and ethno-cultural characteristics of the setting [28]. For example, the economic situation of widespread poverty may contribute to further marginalisation of PMHP who are not able to financially contribute to society [29]. Moreover, the cultural value of collectivism may results in discrimination towards PMHP especially with regarding to marriage and childrearing, since a person’s mental health problem is often seen as the family’s mental health problem [30]. Overall, practices and outcomes of stigma differ across cultures and socioeconomic backgrounds [29, 31, 32], and meaningful comparison across cultural settings may not be achievable with cross-cultural measures [33]. In consideration of this, researchers have called for an in-depth qualitative exploration of the experiences of stigma among PMHP in LMICs settings, where about 85% of the world’s population live [21].

PMHP in the Philippines, a lower-middle income country in Asia, might experience a significant level of stigma and discrimination. Filipino immigrants believed that personal characteristics (i.e. self-centeredness and “soul weakness”) resulted in mental health problems [34, 35], which have been shown to be related to blaming PMHP and discriminatory behaviour in other settings [36]. Also, a multi-country survey revealed that, among 16 countries surveyed, the Philippines had the second highest proportion of citizens who agreed that PMHP should not be hired for a job even if they are qualified [37]. Further, some studies that involved interviews with Filipino immigrants living in Australia and the United States and that sampled from the general population revealed that a fear of being labelled as ‘crazy’ and spoiling their family’s reputation made Filipinos hesitate to seek help from mental health professionals [35, 38, 39]. Although these previous studies provide some knowledge regarding public stigma in the Filipino context, all of them looked at stereotypes, prejudices and intentions to discriminate held by the general public towards PMHP. To our knowledge, there is no study investigating PMHP’s own experiences of being stigmatised and discriminated against and the related factors in the Philippines.

To fill the gaps in the literature, we conducted a qualitative study on the factors related to experiences of stigma as well as the experiences itself of PMHP in the Philippines, using interviews with PMHP and people who know them well. Revealing the existence, types, and sources of stigma experienced by PMHP in the Philippines can contribute to the stigma research in Asian LMIC settings. Further, exploring the experiences of stigma and its related factors can provide fundamental knowledge for the design of an effective stigma reduction program in the Filipino setting.

## Methods

The current research utilised the principles of constructivist grounded theory, which is deemed suitable for revealing the social phenomenon of PMHP’s experiences of stigma [40] in the Filipino context. The constructivist grounded theory assumes a relativist ontology (accepting that multiple realities exist) and a subjectivist epistemology (involving a co-construction of meaning through interaction between the researcher and participant) [41]. It provides a means of studying power, inequality, and marginality [42].

### Setting

Our study was conducted in Muntinlupa, the southernmost city in the Philippines’ National Capital Region. The city has a population of 481,461 as of 2016. The majority comprises Tagalog ethnic groups and professes Christian, primarily Roman Catholic, faith. Households below the food threshold, the minimum income required to meet basic food needs, account for 21.5% of the total in the city [43]. The majority of citizens cannot afford private medical services, which cost five times more than the public medical services [44]. With respect to public psychiatric service, the city has one outpatient and no in-patient facility. The nearest public in-patient psychiatric facility is located about 23 km away.

### Main data collection

#### Participants

We collected data on PMHP from three different sources of information: PMHP themselves, their carers, and community health volunteers who knew them well. The eligibility criteria for PMHP were 1) having a mental health problem, listed in the Diagnostic and Statistical Manual of Mental Disorders 5 (DSM-5), or epilepsy, and 2) currently not using residential care. Epilepsy was included for several reasons. First, people with epilepsy are known to suffer stigma and discrimination [45, 46]. Second, the condition has a long history of being classified as a psychiatric problem [47]. Third, even with the present-day efforts promoting mental health in LMICs, epilepsy is often treated together with mental health issues [48]. Last, pilot interviews revealed that local lay people do not clearly differentiate epilepsy from mental health problems.

For the recruitment, we approached 42 PMHP in person; one of them declined to participate owing to time constraints. Thus, we obtained informed consent from 41 PMHP. Among them, two PMHP were excluded because they were confirmed to have only physical health problems and no mental health problems as listed in DSM-5. Consequently, we used data of 39 PMHP for our analysis. The profiles of the final sample are shown in Table 1. In 20 of the PMHP, we interviewed the PMHP and their main carer, usually a parent or sibling. In the remaining 19 PMHP, only a main carer was interviewed, as the 19 PMHP had communication difficulties that hindered them from answering interview questions. Additionally, in 11 PMHP, we conducted interviews with a community health volunteer who was in charge of the district in which the PMHP lived.

**Table 1:** Profiles of people with mental health problems

	n	*N* = 39 (%)
Sex		
Male	26	(66.7)
Female	13	(33.3)
Age range		
0–19 years	11	(28.2)
20–39 years	18	(46.2)
40–59 years	8	(20.5)
60–69 years	2	(5.1)
Highest educational attainment		
No formal education	4	(10.3)
Primary school or lower	16	(41.0)
High school or higher	17	(43.6)
Still in full-time education	2	(5.1)
Employment status		
Out of work^a^	34	(87.2)
Employed for wages	4	(10.3)
Self-employed	1	(2.6)
Marital status		
Single^b^	32	(82.1)
Married/ Domestic partnership	5	(12.8)
Widowed/ Separated	2	(5.1)
Religion		
Roman Catholic	28	(71.8)
Iglesia ni Cristo	3	(7.7)
Protestant	3	(7.7)
Islam	1	(2.6)
Other Christian	4	(10.3)
Classification of mental health problems		
Neurodevelopmental	13	(33.3)
Schizophrenia Spectrum and Other Psychotic	10	(25.6)
Substance-related and Addictive	8	(20.5)
Epilepsy	2	(5.1)
Anxiety	2	(5.1)
Trauma and Stressor-related	1	(2.6)
Depressive	1	(2.6)
Sleep -Wake	1	(2.6)
Data deficient	1	(2.6)
Lifetime mental health or welfare service use		
Yes	26	(66.7)
No	13	(33.3)
Current mental health or welfare service use		
Yes	12	(30.8)
No	27	(69.2)

^a^Children under 15 years old, the legal working age, (*n* = 3) are included

^b^Children under 18 years old, the legal marriage age, (*n* = 8) are included

#### Recruitment

We aimed to include a wide variation in the characteristics of the PMHP, namely, gender, age, marital status, educational attainment, employment status, religion, type of mental health problem, and history of using health and welfare services. To achieve this, the participants were recruited by purposive sampling in cooperation with two different collaborating stakeholders. First, as stigma was considered to inhibit Filipino people from seeking professional help for their mental condition [35, 49], we recruited the majority of PMHP (*n* = 36) in cooperation with community health volunteers, which enabled us to recruit PMHP regardless of their history of receiving health care. The community health volunteers had good knowledge of the profiles of the residents of the district under their charge and covered all the areas of the city. Second, we recruited a small number of PMHP (*n* = 3) with common mental health problems (e.g. anxiety and depressive problems) from the outpatient clinical practice of a psychiatrist, as the community health volunteers did not identify any people with these types of problems.

To check the eligibility of those who had never been diagnosed by a specialist as having a mental health problem, a research member, ET, carefully reviewed the data of the individual participants, including interview recordings, transcriptions, and field notes, and then provided informed presumption if the participants had a mental health problem or not. ET also assessed which chapter, the broadest classification in DSM-5, the participant most fitted. ET has clinical experience as a psychiatrist in Japan for over 15 years.

#### Interview procedures

Data on the PMHP were collected through semi-structured in-depth interviews. Prior to the beginning of data collection, an interview guide was developed, referring to previous research [18, 50], and then modified based on six pilot interviews in the setting. The interview guide had a series of open questions on three major topics: onset of mental health problems and coping behaviours, experiences of being treated negatively owing to the problem and its consequences, and activities PMHP gave up because of how others might respond to their health problem. The interview guides for interviews with PMHP and for interviews with carers and community health volunteers can be accessed in Additional files 1 and 2, respectively. Consistent with the grounded theory methods, we used the interview guide as a flexible tool that could be revised as the analysis progressed. The carers and community health volunteers were not asked about their own experiences of stigma as a carer or person working in mental health. Instead, we asked them about the PMHP’s experiences regarding the same topics, based on their observations. Demographic data of the PMHP were also obtained at the beginning of the interview.

The first author, CT (female, a Japanese public health nurse), conducted all of the data collection between January and March 2017. During the interview, Tagalog or English was used as preferred by the participants. When Tagalog was chosen, the interviews were interpreted by one of two health workers who had lived in the city for more than 30 years and were fluent in both Tagalog and English. After explaining the study and gaining informed consent, the interviews were conducted in their home, a health centre, or the city hospital, depending on the participants’ preference. Wherever possible, we conducted interviews in a space where there was no one but the interviewee, interviewer, and interpreter around. However, five PMHP were not willing to be interviewed alone. In which case, a family member was in the same place and assisted the interview. All the interviews were digitally recorded with interviewees’ permission and lasted between 19 and 53 min; the median length was 29 min. The participants received 100 Philippine pesos (1.9 US dollars) as acknowledgement for their participation.

### Supplementary data collection

We included data of interviews with seven health workers into our analysis to gain a wider perspective on the stigma experienced by PMHP. CT conducted the interviews during her one-month participant observation at health services provided by the city government. During the observation, CT discussed the role of stigma and its impact on PMHP with more than 85 health and welfare workers. We analysed seven interviews with those who shared episodes on PMHP with whom they were in direct contact as a part of their duty at work. The interviewees were three community health volunteers, two nurses, one doctor, and one rehabilitation program officer. Notes were taken during the interviews and six out of seven interviews were audiotaped with their permission.

### Analyses

All of the recordings were transcribed verbatim by two trained transcribers. Tagalog recordings were simultaneously translated into English by the transcribers fluent in English and Tagalog. An independent research assistant randomly selected 10% of the English transcripts and checked their accuracy by matching them with the Tagalog and English recordings. During this checking process, no significant errors were found thus the transcripts were quality assured.

Data analysis started as soon as the initial data were collected. We set aside theoretical ideas from the existing literature; instead, we remained open to exploring the theoretical possibilities we could discern from the data. After reading each of the transcripts at least twice, CT and ET independently conducted the initial coding. Simple codes were created to describe the phenomenon in each segment of data, using the qualitative data analysis software, Nvivo Version 11.4.1 (QSR International, 2016). The initial codes with identical meanings were merged through discussion, whereas those with different meanings were left unchanged to increase the variety in the interpretation of the data. We used data from interviews with cares, health volunteers, health and welfare workers to increase variety of data on stigma experienced by PMHP and gain comprehensive understanding of its context. Thus, when accounts showed some discrepancy between a person with mental health problem and his/her carer or a person who knew him/her well, we used the data from both accounts for our analysis.

The authors gradually moved on to the focus coding, in which the initial codes were concentrated on or collapsed into categories that make analytical sense, and then tested these against extensive data. The interpersonal interaction between people with and without mental health problems was treated as the central phenomenon of our interest. To explore comprehensively PMHP’s experiences of stigma, we decided to treat any “uncomfortable treatments from others” reported as stigma experience, regardless of the actors’ motivation. We constantly compared data on similarities and differences within a participant as well as across participants to examine the categories and develop links among them. CT led the preliminary focus coding. Subsequently, discussions were held between CT, ET, and HM, in which we reviewed the developed categories and links to determine if they were grounded in data and sufficiently explained the phenomenon.

After analysing the data of the 35 PMHP, a tentative model that explains the relations between categories was developed. We then collected and analysed data on four additional PMHP. Through discussion, the full research team determined that the categories and themes were sufficiently relevant and that the model held true for these additional PMHP. We then concluded that the model was theoretically saturated.

## Results

**Fig. 1:**
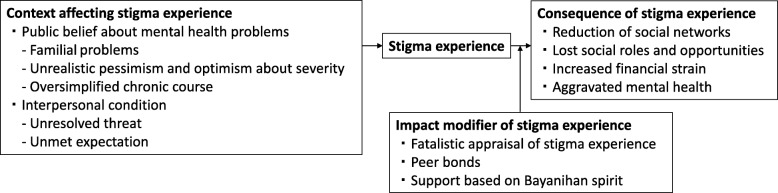
Stigma experienced by people with mental health problems and its related factors in the Philippines

Analyses revealed four interrelated themes surrounding stigma experienced by PMHP: (1) the context affecting stigma experience, (2) stigma experience, (3) impact modifier of the stigma experience, and (4) consequence of the stigma experience. Figure 1 shows the relationship among the themes.

### Context affecting stigma experience

We identified two contextual categories that changed how others treated PMHP in a negative way.

#### Public belief about mental health problems

Public beliefs surrounding mental health issues are a contextual category of stigma experienced by PMHP in the Philippines. It consists of three themes: familial problems, unrealistic pessimism and optimism about severity, and oversimplified chronic course.

#### Familial problems

Community health volunteers and health workers observed that families of PMHP and people in the local community do not provide appropriate support for PMHP because they perceive mental affliction as a family problem and indicative of so-called “bad blood”. The belief that mental health problems can be transmitted among relatives pushed families of PMHP to deny the existence of mental health issues and people in the community to distance themselves from PMHP. A nurse shared an episode of a male patient with depression:

*His family could not accept the idea that one of their relatives is actually depressed. (…) It’s because in our culture, when it comes to mental illness, it tends to be a family affair. People think if one of you has a history of mental illness, there is a chance that almost all of you already have that as well. We care about how others think about our family more than anything else. And other people feel that it is not their place to intervene in some family matters. (Interview 48, Nurse, Female)*

In particular, marrying age PMHP faced stigma because of the belief in heredity. People in the community often believe that PMHP have mental health problems in their family’s blood and are afraid of developing those problems in their kinship via marriage.

*I had one neighbour that I reported to the barangay [district government] because she mocked me. She was saying that I had mental illness in our blood and no one dare marry me and get in trouble. (Interview 51, PMHP, Male)*

#### Unrealistic pessimism and optimism about severity

PMHP experienced stigma when others were overly pessimistic about the severity of a mental health problem. Participants often criticised those who believe that mental health problems generally cause severe functional impairments. This belief has resulted in unfair treatment towards PMHP in the Philippines.

*[Researcher: What is the biggest challenge for the [social inclusion] program?] Finding a job. It’s very difficult. The community people don’t believe they [PMHP] are functional and don’t hire them. (…) So now some barangays [district governments] have started to hire them. We hope people see them working hard and start to trust them. (Interview 27, Rehabilitation program officer, Male)*

Meanwhile, unrealistic optimism about its severity also caused stigma. The commonly held belief is that individuals are able to overcome any psychological suffering by themselves, and as a result, it will not become a serious problem. It was common for PMHP to be doubted or withheld empathy in such a culture that emphasises resilience and humour under stressful situations.

*Filipinos are proud of being resilient. We find something funny in any difficult situation. But when you have this illness, that kind of thinking gives you a huge pressure. (…) One day, I opened up about my mental illness to my friends, but they all had the same reaction. They laughed at me and didn’t take it seriously. (Interview 71, PMHP, Female)*

#### Oversimplified chronic course

The *oversimplified chronic course* of mental health problems emerged as one of the causes of stigma. People without any experience of a mental health problem often misunderstand the repetitive relapse and remission in the course of a mental health problem. They tend to apply an acute illness model and expect a complete cure in the short term. However, as the symptoms are prolonged, they begin to mistrust the PMHP’s account.

*After one month of no work, I was able to work and sleep. But in February, it came back. I couldn’t sleep for several days. (...) My supervisors were thinking that I should be working a night shift duty, but I told them that I would have to take sick leave. But because it was the same reason for my previous absent, they are already thinking that I am making up stories. (Interview 30, PMHP, Male)*

#### Interpersonal condition

*Interpersonal condition* was identified as a direct trigger of stigma experience. It consists of two themes: unresolved threat and unmet expectation.

#### Unresolved threat

*Unresolved threat* is a condition where PMHP are at risk of hurting themselves or others owing to their mental health problems, with the people in contact with the PMHP failing to manage such risks. Under such conditions, PMHP often experience physical violence, being avoided, and being restricted by others. Although the PMHP, their families, and community health volunteers attributed the threats to PMHP’s personal factors, such as personality and outwardly noticeable symptoms, they also emphasised the culpability of people in the local community for their lack of understanding and skills in interacting with PMHP. When others became familiar with PMHP, they successfully managed those threats and prevented PMHP from experiencing stigma. The mother of a boy with a neurodevelopmental problem told us:

*My son easily becomes violent. For example, when someone takes and plays with his toy. The neighbours don’t understand why he is angry and they bully him. But there are also some playmates who fully understand him. When they know that my son is about to be angry, they immediately keep distance from him. And after a while, my son calms down and they start playing around together. (Interview 4, Mother of a boy with a mental health problem)*

#### Unmet expectation

*Unmet expectation* was another context of stigma. In this context, there is a gap between PMHP’s abilities and other people’s expectations of them. Some PMHP reported suffering from stigma when others’ expectations were too high for their situation. People in this cultural setting tend to value strong bonds and reciprocity among families and neighbours. PMHP sometimes were unable to perform in accordance with this value owing to their mental health conditions. Violation of this value was judged as morally wrong.

*They [the neighbours] say I should help my mum by doing washing, cleaning, and taking care of my brother, even when I say I feel weak or don’t know how to. (Interview 5, PMHP, Female)*

*She is big but still doesn’t help her mother. That’s why the neighbours don’t like her. They say she is not a good daughter. (Interview 18, Community health volunteer, Female)*

Meanwhile, some other PMHP experienced stigma when others underestimated PMHP’s abilities. Families often criticised other people that looked only at PMHP’s disabilities but not at their abilities.

*When someone in our neighbourhood was trying to talk to my sister and she did not respond back, they started bullying her and calling her crazy. [Researcher: How do you think we can change such situation?] I think proper communication towards her would be the best since she’s really a good listener. The problem is that other people don’t know she actually understands things really well. (Interview 8, Sister of a woman with a mental health problem)*

### Stigma experience

Although we frequently found that PMHP were positively treated by others because of their mental health problems, we also discovered that almost all the PMHP participants were faced with negative treatment from others. PMHP experienced *psychological abuse* (e.g. being verbally insulted, laughed at, stared at, gossiped about, doubted), *physical violence* (e.g. being hit, stones being thrown at them), being *restricted* (e.g. being told not to go outside alone, tied with a rope to a pillar), not being *supported* (e.g. lack of understanding and sympathy), being *taken advantage of* (e.g. being cheated out of money and belongings), being *neglected* (e.g. privacy not being protected, medical care not being provided), and being *rejected* (e.g. not being associated with, not being hired). Families were an important source of stigma in terms of prominence as stigma from families was often repetitive (e.g. frequently being slapped) and prolonged (e.g. being locked up in a room for several months). PMHP also experienced stigma frequently from their neighbours, and sometimes from school friends, co-workers and employers. People who were involved with PMHP as a part of their duty at work (i.e. health workers and public safety officers) were a source of stigma as well. For complete information on the stigma experience by source, please see Table 2.

**Table 2:** Stigma experience by type and source and its examples^a^

Psychological abuse	
Family	My other siblings say that she’s crazy. (Interview 70, Sister of PMHP)
Friends	Her classmates were bullying her (Interview 21, Mother of PMHP)
Neighbours	They [neighbours] say bad things to me like “abnoy” [“abnormal” in Tagalog]. (Interview 55, PMHP)
Coworkers, Employers	I don’t like them [coworkers] gossiping about me. (Interview 32, PMHP)
Health care providers	They told me that I was lazy. (Interview 42, PMHP)
Strangers on the street	When a vehicle stopped and the driver stared at her I got mad. (Interview 38, Father of PMHP)
Physical violence	
Family	He [Father] sometimes slaps him. (Interview 15, Mother of PMHP)
Friends	My high school friends started throwing stones at me and saying I’m crazy (Interview 6, PMHP)
Neighbours	When my neighbour hit him I needed to bring him to the hospital (Interview 1, Mother of PMHP)
Public safety officers	They [public safety officers] hit me and I was very scared. (Interview 58, PMHP)
Restricted	
Family	We tied him down with a rope because he would always wander around. (Interview 43, Mother of PMHP)
Not supported	
Family	Her brothers don’t understand her. (Interview 8, Sister of PMHP)
Friends	No one really understands me, even my friends. (Interview 60, PMHP)
Coworkers, Employers	They [coworkers] usually laugh because they don’t know it’s hard having this problem. (Interview 12, PMHP)
Taken advantage of	
Neighbours	He usually rents a bike in our neighbour for five pesos, but sometimes he’ll pay with twenty pesos and they won’t give him the change. (Interview 7, Mother of PMHP)
Neglected	
Family	We had to let him live with us because his parents abandoned him when he was little. (Interview 44, Aunt of PMHP)
Health care providers	When we visited the hospital, we saw that she was naked and she was already held with other mental patients in the room. There were no assessments done for her [for urinary tract infection]. (Interview 38, Father of PMHP)
Rejected	
Family	My brother says to “keep her out of the home.” (Interview 63, Sister of PMHP)
Friends	[Researcher: Did he have many friends?] Yes, before. But now, they avoid him. (Interview 65, Mother of PMHP)
Coworkers, Employers	I kept failing to find a job. That’s why I need to hide it [mental health problem]. (Interview 16, PMHP)

^a^A listed source with an example of a particular type of stigma experience indicates that at least one incidence of that type was reported by the participant

### Impact modifier of stigma experience

Even if the nature of stigma experiences were similar, the extent and degree of its influence on PMHP’s life varied depending on *impact modifier of stigma experience*. PMHP had three impact modifiers consisting of internal (i.e. fatalistic appraisal) and external (i.e. peer bonds, community unity) factors.

#### Fatalistic appraisal of stigma experience

*Fatalistic appraisal of stigma experience* offered PMHP and their families a strategy to cope with the emotional pain caused by stigma experience. People in the setting generally believed that God predetermined life events in the past, present and future. Some PMHP and their family accepted unfair treatments from others as “fate.” They were able to remain hopeful because they believed that God would help them if they had faith in God.

*Sometimes people say he is crazy. [Researcher: What do you do in response to that?] Nothing. People say what they want to say. We just say “God is good.” As long as we believe in Him, it will be alright. (Interview 23, Sister of a man with a mental health problem)*

#### Peer bonds

*Peer bonds*, the emotional bonds with other people with similar mental health problems, empowered PMHP to change their stigmatised situation in a positive way. Stigma experience could marginalise them in the community, but when they were together with peers who understood not only their health condition but also their lowered social status, they were empowered and motivated to change the situation for themselves and their peers.

*[Researcher: What are the barriers to your recovery?] The different perceptions of people towards us [she and other people with mental health problems]. It is so discouraging for us. And we are the only ones who can understand each other very well. We are like brothers and sisters already. Nevertheless, we make sure that the reason we join the [rehabilitation] program is not only for ourselves but to show them that we can change ourselves for the better. If we will be given a chance to work again, we will make 100 percent effort to get things done accordingly. (Interview 34, PMHP, Female)*

#### Support based on Bayanihan spirit

*Support based on Bayanihan spirit*, a traditional concept of community unity, relieved the negative impacts of stigma on PMHP. It was not rare that community people gave food or rented a house free to PMHP and their family who had little income. Helping one another in a time of need was inherent in their lives, called Bayanihan in Tagalog. For example, a homeless woman with schizophrenia told us that she had felt hopeless because she had been bullied at school and was in a materially deprived circumstance. However, she was now enjoying her life and managing to make a living because some of her neighbours treated her as a valued community member (e.g. regularly invited her to a local dancing event) and occasionally gave her food. A community health volunteer explained why she had good relationships with the community as follows:

*That is natural here. When your family member is sick, neighbours and friends are there to pay for medicines, bring food, help with housework, and take care of small kids. We call it Bayanihan. (Interview 3, Community health volunteer, Female)*

### Consequence of stigma experience

Stigma experience was found to bring about a substantial negative impact on PMHP’s social networks, roles, opportunities, and mental health.

#### Reduction of social networks

Stigma experience reduced PMHP’s social networks, which led to them spending their days isolated at home without any interaction with people outside of their immediate family. This was due not only to the direct influence of experiences of stigma (i.e. being *physically restrained*, being *avoided by others*) but also the indirect influence of changes in three aspects: PMHP’s behaviour, restriction by families, and relationships with others. First, after being negatively treated, PMHP tended to “close off to everybody” and distanced themselves from others.

*Going out is sometimes like an obstacle. (…) After that [hearing my friends gossiping about me], I have been afraid of people’s judgments. (Interview 62, PMHP, Female)*

Second, families started to restrict PMHP’s behaviour to protect them from further stigma experiences.

*We do not allow him to go out. We are afraid that something like that [neighbours calling him crazy] might happen to him again or someone might abduct him. (Interview 9, Sister of a man with a mental health problem)*

Third, stigma experiences provoked conflicts, from a quarrel to a physical fight, and worsened the relationship between PMHP and others. The conflicting relationships produced a further stigmatising attitude towards PMHP.

*He got into a fight with his playmates because they said bad words to him. (…) Many of our neighbours told me that he should be in a cell. They told me that they knew a policeman who could put him in jail. (Interview 1, Mother of a man with metal health problem)*

#### Lost social roles and opportunities

As a result of stigma experience, PMHP *lost social roles and opportunities*, such as being employed, going to school, having a romantic partner, getting married, parenting, helping with household chores and the family business, taking care of younger siblings and joining religious activities.

*She was a member of the choir in church. She likes singing and has a good voice. And plenty of friends visited her in the past and they went to church together. But no more. Nobody visits her, and she quit attending it. (Interview 10, Mother of a woman with a mental health problem)*

#### Increased financial strain

Lost social roles and opportunities increased financial strain, which negatively affected the families as well as PMHP themselves. In this setting, PMHP and their families lived in communities where many people find it difficult to make a living. The cost of transportation to medical facilities and treatment fees put them in a further difficult situation economically. In such conditions, entire families often suffered from the financial strain that was due to stigma to the degree that they could not afford basic items including food and clothing.

*If only I could find a good job like when I was well. Even though we do not have enough money to buy things, my family really makes an effort to find ways that we can buy those medicines. (Interview 20, PMHP, Male)*

#### Aggravated mental health

The participants reported that the stigma experiences *aggravated mental health* in PMHP. The memory of negative treatment from others often stuck in their mind and its influence lasted for a long time. A 32-year-old woman with anxiety problem explained how the experience of being bullied when she was a teenager influenced her current condition:

*It triggers my anxiety. When I remember their facial expressions, even now, I feel overwhelmed and breathless (Interview 39, PMHP, Female).*

The experience of stigma also affected the mental health condition of PMHP by preventing them from seeking help. Some PMHP and their families choose to keep their mental health status a secret. However, families have limited capacities to take care of a person with a mental health problem, especially in the case of someone with severe symptoms. In the worst case in terms of the influence of stigma on PMHP’s mental health, a community health volunteer reported that the parents of a daughter with a mental health problem locked her up in her room and took care of her without seeking professional help. However, her condition kept deteriorating and eventually she committed suicide inside her room.

## Discussion

To our knowledge, this is the first study to document the stigma experienced by PMHP in the Philippines. This study adds to the understanding of discrimination in LMIC settings and its related contextual factors in the Philippines.

First, our results showed that PMHP in the Philippines experienced stigma, which brought about negative impacts on PMHP’s social networks, roles and opportunities, financial burden, and mental health. Although stigma types, sources, and areas of impact were generally consistent with the existing literature in this field [4, 6, 51], we found that experiences of stigma threatened the economic survival of the entire family of PMHP and increased the mental health crisis in the LMIC context, given the minimal welfare and mental health care provisions. Several studies with participants recruited from clinical settings have shown that PMHP in LMICs suffered less from stigma [2, 18–20]. In this study, we involved PMHP without psychiatric service use, which prevented us from overlooking the stigma experienced by the poorest and most marginalised PMHP. Our findings might better reflect the reality in LMIC settings, where it is estimated that more than 70% of PMHP receive no treatment for their mental health conditions [52].

Second, we found that pessimistic and over-optimistic reactions to a mental health problem are among the important contexts of experiences of stigma in the Philippines. Historically, stigma research has mainly focused on the pessimistic view on the prognosis and its negative effects [10, 53–56]. Meanwhile, when the over-optimistic view on the outcome of mental health problems has been documented among Filipino immigrants, it was only recognised as a barrier to help-seeking [35, 39]. Our qualitative exploration’s original finding is that the over-optimistic belief among the community regarding the severity of mental health problems results in PMHP’s receiving inappropriate or negative treatment. This is an important finding for the Philippines, because resilience and optimism under difficult situations are among the well-known cultural traits of Filipinos [57, 58]. Stigma resulting from optimism might be prevalent in the Philippines; a prior study showed that among the 16 countries, the Philippines posted the highest proportion of respondents who agreed that mental illness would improve on its own [59].

Third, the results indicated that mental health problems were perceived as problems of the family and discouraged people from accepting mental health problems. The finding is consistent with psychiatrists’ clinical experiences with Filipino patients [60, 61]. We also found that a belief in transmissibility among relatives led to PMHP experiencing reduced marriage opportunities. Previous studies conducted on Chinese descent groups [62–64] showed that the threat of genetic contamination was related to endorsement of reproductive restriction. We propose that it might hold true in the Filipino context, meaning that the threat to family lineage through genetic contamination via marriage accounts for some of the discrimination experienced by PMHP.

Fourth, we revealed a context-specific impact modifier of stigma experiences, namely, *fatalistic appraisal of stigma experience*. Existing studies have discussed that Filipinos typically attribute illness to “the will of God” [39, 49, 65]. A new finding of this study is that negative treatments from others were also attributed to fate. Globally, it is known that fatalistic appraisal of negative events inhibits active coping and worsens health [66, 67]. However, we found that fatalism offered a spiritual coping strategy and shielded PMHP from the adverse effects of stigma in the Catholic dominant setting of the Philippines. These findings are consistent with the literature that have showed that fatalism facilitates adjustment to negative life events [49, 68, 69]. Moreover, *support based on Bayanihan spirit* was another culturally relevant impact modifier. The origin of the Bayanihan spirit is traced back to the country’s tradition wherein towns’ people cooperate to carry a family’s entire house on their shoulders to a new location. It is considered a core essence of the Filipino culture. Our finding supports the arguments by Lasalvia [21] and Mascayano et al. [29] that communal network, which tends to be better maintained in LMICs, is among the existing strengths to reduce the negative effects of stigma.

Lastly, the research method of obtaining perspectives from multiple participants who witnessed and experienced stigma allowed us to reveal that the interpersonal conditions (i.e. *unresolved threat* and *unmet expectations*) preceded stigma experiences. Consistent with previous research from India [24] and Indonesia [70], in the setting where mental health care is not readily available at a local level, people in the community needed to cope with the possible danger of PMHP to self or others and can violate PMHP’s human rights. Similar to the results of prior qualitative analyses of interviews with PMHP and their families [18, 71], the expectations of others in contrast to PMHP’s actual capabilities caused negative reactions from others. Those interpersonal conditions might be a more important determinant of stigma experiences than PMHP’s personal factors, considering the previous studies showing individual variables (e.g. employment status, symptom, and treatment experiences) accounted for only less than 30% of total variance of experienced stigma [2, 3].

### Practical implications

Our results suggest that mental health care must have the objective of the reduction of stigma towards PMHP. The Department of Health and Local Government Units are required by the Mental Health Act [72], established in 2018 as the first law of its kind in the Philippines, to initiate and sustain nationwide campaigns to raise the level of awareness on the protection and promotion of mental health and rights. In conducting stigma reduction campaigns, they should: 1) target families of PMHP, community people, health workers, and public safety officers; 2) avoid genetic explanations for mental health problems and emphasise the role of environmental and social factors as its cause; 3) increase public understanding of not only the possibility of recovery but also the challenges that PMHP face; and 4) improve families’ and community members’ skills in assessing and coping with possible danger posed by PMHP to self or others [73–76]. These interventions might be more effective when they utilise the existing communal network and increase social contact between PMHP and others [77, 78] We also propose that mental health and welfare services for PMHP should: 1) be community-based and support PMHP in meeting expectations that are meaningful for themselves and others; 2) provide opportunities for PMHP to share their experiences with peers to empower them [79–81]; and 3) prevent PMHP from internalising experiences of stigma with acknowledgement of fatalistic appraisal of them as a coping strategy. Lastly, to mitigate the adverse influences of stigma, it is necessary to change the structure of health care and welfare service provision for PMHP (e.g. inclusive education, welfare benefits, and job schemes). It is also essential to provide effective and accessible mental health care.

### Study limitations

We were unable to recruit people with common mental health problems who were not using psychiatric services. In fact, community health volunteers do not recognise any people having common mental health problems. This may reflect stigma-related situations where local people do not recognise the manifestation of symptoms of those problems as a health issue, or where people with those problems hide their conditions. Additionally, cultural and language barriers may have played a part in data collection and interpretation. However, we also encountered a number of situations where the interviewee provided the data collector, who was from another cultural background, with further explanations, especially on their culture. Further, some interviews were too short to be considered an in-depth interview. Also, we needed to rely in part on data from narratives of people who know PMHP well, instead of from PMHP themselves. These were because the interviewer had difficulty encouraging some participants, especially PMHP, to talk about sensitive topics. Thus, there might be experiences and related themes that we could not explore. Lastly, we conducted the study in one city; thus, the results may not be generalisable to another part of the Philippines (e.g. rural and Muslim-dominant areas).

## Conclusions

Our findings highlight that PMHP in the Philippines experience substantial discrimination and its adverse effects are severe to the degree that it threatens the financial survival of the entire family. Culture-bound beliefs and social structure (e.g. perceiving mental health problems as a familial problems, traditional communal unity) played important roles in shaping and modifying stigma experiences. More research is needed to develop stigma reduction interventions utilising these findings and to evaluate their effectiveness.

## Additional files

Additional file 1: Interview guide for interviews with people with mental health problems. A set of questions we referred while interviewing PMHP. (DOCX 88 kb)

Additional file 2: Interview guide for interviews with carers and community health volunteers. A set of questions we referred while interviewing carers and community health volunteers. (DOCX 90 kb)

## Abbreviations

DSM: The Diagnostic and Statistical Manual of Mental Disorders
HICs: High-income countries
LMICs: Low- and middle-income countries
PMHP: People with mental health problems
