# Supplementary material for: A qualitative study on the stigma experienced by people with mental health problems and epilepsy in the Philippines
Source: BMC Psychiatry. 2018 Oct 5;18:325. doi: 10.1186/s12888-018-1902-9 (PMC6173886; doi:10.1186/s12888-018-1902-9)
Supplement: Supplementary file 1 — Interview guide for interviews with people with mental health problems. A set of questions we referred while interviewing PMHP. (DOCX 88 kb) [file 12888_2018_1902_MOESM1_ESM.docx]

**Additional file 1**

**Interview guide for interviews with people with mental health problems**

Introduction

I would like to start by asking you a little about yourself.

- Can you tell me what your day is like?

(Who is at home, occupation, routine, interests, social network, who he/she spends a lot of time with)

Illness onset and coping behaviours

Next, can you tell me about the psychological/ emotional/ mental health problems you have?

- When did you first notice these problems?
- What did you do then? (establish process of help-seeking, approximate course of illness)
- What do you think has caused these problems?

Experiences of being treated negatively

Now I’d like to ask you about your daily life before and after these problems started.

- Have these problems changed relationships between you and others?
- What has changed?
- Can you give me examples? (How is it now? How was it before these problems started?)
- Would you prefer some people not to know? Who should not know? Why?
- How do ___ （※）treat you? Can you give me examples?
- How do you feel about it?
- How would you like ___ treat you?
- If it seems difficult to answer these questions, raise the following relating people as examples.
- Family members
- Relatives
- Friends
- Intimate partner
- Colleagues
- Neighbors
- People at church, temple, mosque
- People in street or on bus
- Health care providers

Activities people with mental health problems gave up

- Have others discouraged or stopped you from doing something because of these problems?
- Can you give me examples?
- How do you feel about it?
- Have others encouraged or persuade you to do something because of these problems?
- Can you give me examples?
- How do you feel about it?
- Have you ever hesitated or gave up doing something because of these problems?
- Can you give me examples?
- How do you feel about it?
- If it seems difficult to answer, raise the following life domains as examples.

　　Probe for life domains:

- Life at home (housekeeping, earning money, parenting, decision making, meeting visitors, going out, doing religious rituals)
- Gatherings with relatives (seeing them, attending family celebrations)
- Friendships (getting in touch with them, joining recreational activities)
- Marriage (getting married/divorced)
- Education/ work (joining a school, keeping a job, finding a job)
- Politics (voting, attending political activities)
- Activities in neighborhood (attending community gatherings, religious worships)

Closing questions

We have reached the end of the interview.

- Is there anything more you’d like to tell me that we have not already discussed?
- Do you have any questions about anything we discussed?

Thank you again for your time and for allowing me to talk to you.
