# Supplementary material for: A qualitative study on the stigma experienced by people with mental health problems and epilepsy in the Philippines
Source: BMC Psychiatry. 2018 Oct 5;18:325. doi: 10.1186/s12888-018-1902-9 (PMC6173886; doi:10.1186/s12888-018-1902-9)
Supplement: Supplementary file 2 — Interview guide for interviews with carers and community health volunteers. A set of questions we referred while interviewing carers and community health volunteers. (DOCX 90 kb) [file 12888_2018_1902_MOESM2_ESM.docx]

**Additional file 2**

**Interview guide for interviews with carers and health volunteers**

Introduction

Could you tell me about the relationship with XX?

- When did you first meet XX?
- How often do you meet or contact XX recently?

Illness onset and coping behaviours

- When did you first notice XX’s emotional/ psychological/ mental health problems? How?
- How did you feel?
- What did you say to XX at that time?
- What do you think has caused XX these problems?

Experiences of being treated negatively

Now I’d like to ask you about relationships between XX and others before and after XX started to have these problems. Please answer my questions as far as you know.

- Have XX’s problems changed relationships between XX and others?
- What has changed?
- Can you give me examples? (How is it now? How was it before these problems started?)
- Do others(※) know about XX’s problems?
- Do you think XX prefers some people not to know? Who should not know? Why?
- How do others treat XX? Can you give me examples?
- How do you think XX feels about it?
- How do you think XX would like ___ treat him/her?
- If it seems difficult to answer these questions, raise the following relating people as examples.
- Family members
- Relatives
- Friends
- Intimate partner
- Colleagues
- Neighbors
- People at church, temple, mosque
- People in street or on bus
- Health care providers

Activities people with mental health problems gave up

- Have others discouraged or stopped XX from doing something because of XX’s problems?
- Can you give me examples?
- How did XX feel about it?
- Have others encouraged or persuade XX to do something because of XX’s problems?
- Can you give me examples?
- How did XX feel about it?
- Has XX ever hesitated or gave up doing something because of XX’s problems?
- Can you give me examples?
- How did XX feel about it?
- If it seems difficult to answer, raise the following life domains as examples.

　　Probe for life domains:

- Life at home (housekeeping, earning money, parenting, decision making, meeting visitors, going out, doing religious rituals)
- Gatherings with relatives (seeing them, attending family celebrations)
- Friendships (getting in touch with them, joining recreational activities)
- Marriage (getting married/divorced)
- Education/ work (joining a school, keeping a job, finding a job)
- Politics (voting, attending political activities)
- Activities in neighborhood (attending community gatherings, religious worships)

Closing questions

We have reached the end of the interview.

- Is there anything more you’d like to tell me that we have not already discussed?
- Do you have any questions about anything we discussed?

Thank you again for your time and for allowing me to talk to you.
